# Enhancing Soldiers for Future Warfare: Good Science; Bad Ethics?

**DOI:** 10.1007/s11948-025-00573-w

**Published:** 2025-12-15

**Authors:** Nicholas G. Evans, Michael L Gross, Ryan Shandler

**Affiliations:** 1 University of Massachusetts Lowell Massachusetts, Lowell, USA; 2 University of Haifa, Haifa, Israel; 3 Georgia Institute of Technology, Atlanta, USA

**Keywords:** Enhancement, Enhanced warfighters, Military ethics, Experiment

## Abstract

Ethical concerns dog emerging technologies designed to enhance warfighter performance. Brain-computer interfaces, exoskeletons, and mind- or body-altering drugs raise fears about risky, invasive, and experimental medical procedures that offer armies physically and cognitively superior soldiers that will dictate and disrupt the course of future war. What counts as enhancement, however, has been subject to longstanding and passionate debate. This study aims to put an end to this dispute by employing a conjoint experimental design to survey a group of military and professional experts from across the world to explore how definitional perceptions of enhancement influence ethical acceptability. Two main findings emerge. First, we find that there already exists a broad agreement about what constitutes enhancement, and this consensus spans countries, discipline, political orientation, and age. Future policy may now be able to accommodate a definition of enhancement that is widely shared among members of the international community. Second, across the board, ethical acceptability diminishes as medical technologies aim for transhuman warfighting capabilities. Enhancement research and development for military purposes must navigate the conflicting ethical demands of medical experimentation and lawful war. Human enhancement is not morally unacceptable but ethically precarious, requiring regulation, oversight, and transparency.

**Supplementary Information:**

The online version contains supplementary material available at 10.1007/s11948-025-00573-w.

## Introduction

For a half-century, Emerging biomedical technologies have generated concerns about human enhancement ethics. Particularly in the context of military medical interventions that radically improve human capacities,^1^ bioethical analysis has raised concerns about the enhancement of warfighters, including the development of “super soldiers” and their effect on military effectiveness and cohesion,^2^ and democratic norms.^3^

What counts as enhancement, however, is a longstanding debate in the bioethical and policy literature. A common definition of enhancement is the increase of a physical or cognitive capacity above a species-typical norm (Evans et al., 2021). The use of modafinil, for example, which allows pilots and other personnel to maintain vigilance and wakefulness for periods of 60 h without the side effects of amphetamines, is often slated as a poster child of enhancement technologies: humans can go for 60 h without sleep, without pharmacological enhancements, but their cognition severely degrades.

Radical enhancements might include the addition of capacities that humans, by their evolution or other natural histories, simply do not have. A US Defense Advanced Research Projects Agency (DARPA) program to investigate the limits of human metabolism, for example, sought to develop a “switch” that would allow humans to shift from relying entirely on food to relying entirely on body fat for metabolic fuel, something well outside our evolutionary history (DARPA, 2003). The installation of brain-computer interfaces to enable communication between humans and artificial intelligences, likewise, extends human capacities far beyond biological possibility (Gordon & Seth, 2024; Miranda et al., 2015).

Other definitions, however, are deflationary in virtue of the relationship they hold between enhancement and outcomes such as well-being. Some definitions of enhancement hold, for example, that enhancements are simply biological or cognitive interventions that make someone’s life substantially better than they otherwise would, where disability is conceived of as the congenital or acquired converse of enhancement (Buchanan, 2011). But on these definitions, Olympic training programs might count as enhancements, as might Ivy League education. Notably, anabolic steroid users are colloquially referred to as “enhanced”, juxtaposed to “natural” weightlifters and bodybuilders, without necessarily exceeding the biological or species-typical limits of strength–though they reach those limits much faster and with a greater chance of success. Finally, tying enhancement to individual outcomes means that what counts as enhancing is judged in terms of its consequences: making humans more intelligent is *only* enhancing insofar as it makes us better off, but if smarter people are less happy, they are arguably *diminished.*

Despite the proliferation of definitional disputes in the philosophical and ethical literature, little work has been done on identifying whether any consensus exists as to whether particular technologies constitute enhancements or not, and the permissibility of these interventions as a function of their status as enhancements. The first of these is particularly important, given that enhancement among other novel biotechnologies can sometimes be objected to on deep-seated reservations about intervention in human biology: the “wisdom of repugnance” (Kass, 1997). While the above formal definitions for enhancements describe various relationships between human physical and cognitive capacities, population- and species-typical function, and the potential of novel technologies to improve human welfare, we remain in the dark as to how individuals conceive of these relationships. This literature is even more sparse in the military, where—arguably—radical human enhancement is likely to find its first applications.

The literature that does exist, moreover, does so with limited scope. A 2022 Pew Research poll noted that 33% of US respondents thought robotic exoskeletons would be a “good idea for society,” and 30% of respondents thought the same of gene editing technologies, but only 13% of respondents thought of brain-computer interfaces as societally beneficial (Rainie, 2022). The same survey found that most respondents in each case thought these interventions would leave people only as well off as they were before, or worse off. However, respondents were only asked about three discrete scenarios: noninvasive exoskeletons that significantly increased strength, germ-line gene editing to reduce the incidence of serious disease, and brain-computer implants for information processing. The second of these, moreover, is only controversially “enhancement” in the extant bioethical literature over the last 40 years.

A 2022 study conducted with students at the UK Joint Services Command and Staff College at Shrivenham found moderate support for enhancements in the military, but only across three kinds of enhancements – pharmacological interventions, neural implants that improve cognitive abilities, and neural devices to power prosthetic limbs (neuroprosthetics) (Sattler et al., 2022). Moreover, it limited its covariate analysis to the location of home military service, military component (land, sea, or air forces), medical branch inclusion, cohort in the College, and number of deployments. It did not include substantial numbers of defense civilian and/or scientific personnel, national security experts, and other stakeholders. Moreover, it used a single established view of enhancement rather than soliciting views from participants about what constituted enhancement.

In response to this, our research aims to understand which factors cause a technological intervention to be viewed as an enhancement, and explore its relationship to the ethics of enhancement, with a focus on military contexts. We adopted a conjoint survey design to create a series of vignettes that, randomized within our sample, allowed the assessment of different features of the enhancement debate to establish individuals’ definitional preferences and their views on the ethics of enhancement. We conducted this research over the Spring of 2024, among an international sample of military medical ethics, national security, emerging technology, and ethics professionals in civilian and military employment. The international scope and professional reach of this sampling exercise sets this study apart as the foremost empirical investigation of elite attitudes toward enhancement policies, and sets a new benchmark to test where international norms sit on this matter, within and across countries.

In fields where conceptual boundaries remain unsettled, epistemic convergence among domain experts offers a valuable, empirically grounded reference point. While such convergence does not dictate how a concept ought to be defined for all ethical, legal, or philosophical purposes, it can help clarify contested terrain and support more coherent dialogue across disciplines. Especially in the case of human enhancement, where competing definitions abound, tracing where experts already align offers a meaningful contribution. As such, our expert survey does not claim to settle the conceptual debate or to replace philosophical theorizing. Rather, it complements and advances the discourse by revealing how the community of specialists interprets the category of enhancement in practice.

This kind of conceptual mapping also has direct relevance for public policy and military strategy. At the research / development stage, the ethics of enhancement align with longstanding clinical principles guiding medical experimentation. Dominant themes include informed consent, exploitation of soldiers who are the subject of enhancement research, and the short and long-term costs and benefits to the research subject and society. Once enhancements are implemented in operational settings, ethical considerations shift toward questions of compliance with international law, oversight, control of enhanced warfighters, unit cohesion, and post-discharge re-integration into civil society (Shereshevsky, 2020; Puscas, 2018; Harrison Dinniss & Kleffner, 2018; Manjikian, 2018). Across these phases, definitional clarity plays a pivotal role in determining when heightened ethical review is warranted or when specific regulatory thresholds are triggered. In this context, identifying patterns of agreement among experts can support more consistent and justifiable policy judgments.

As a conjoint experiment, this study can only explore a limited number of ethical attributes. Focusing principally on the research and development stage of enhancement technology, these attributes include therapeutic and augmentation value, medical risk and invasiveness, reversibility, military use, and technology taxonomy (neurological, wearable, pharmaceutical, and lifestyle). Despite advances in therapeutic brain-computer interfaces to control prosthetics or restore communication capabilities, DARPA rejected irreversible surgically implanted BCIs for military purposes, declaring, “For the military’s primarily able-bodied population to benefit from neurotechnology, *nonsurgical interfaces are required”* (DARPA, 2019). Reservations like DARPA’s put technologically rich military enhancements and the ethical imprimatur required for research and development on a collision course (Canli et al., 2007). This study investigates the tension between enhancement and ethics directly to determine whether and how it justifiably segregates military from medical science or hamstrings the development of vital military technologies.

## Materials and Methods

To examine which combination of features causes military medical interventions to be perceived as ethical enhancement technologies, we conduct a ratings-based conjoint experiment. This research framework has participants evaluate randomly generated scenarios with interchangeable attributes to determine --- without asking them directly --- which features guide their assessment that any particular intervention constitutes an enhancement technology.

**Fig. 1:**
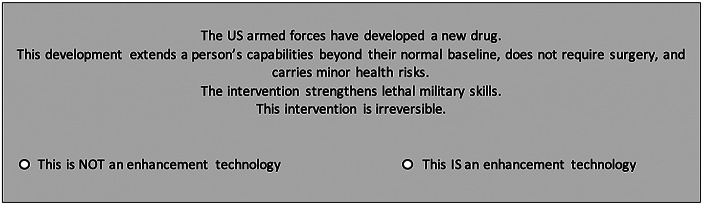
Example of a randomly generated scenario

Each attribute, outlined in Table 1, has between two and four values randomly selected to construct our experimental scenarios. For example, the *type of intervention* might be a ‘mechanical exoskeleton’ or a ‘new drug’, while the *degree of invasiveness* might require ‘major surgery’ or ‘no surgery’. In all, 864 unique scenario permutations could be assembled by drawing one item from each of the six attributes. After viewing a randomly generated scenario, survey respondents answered two questions: “Does the scenario describe an enhancement technology?” and “Is the intervention ethically acceptable?” An example of a randomly generated scenario appears in Fig. 1.

### Survey Instrument

Our conjoint experiment manipulates six attributes, which, when combined, describe a physical intervention that may or may not classified as an enhancement technology. Each attribute is listed in Table 1 and discussed in the following text.

First, we manipulate the *type of intervention*. While the items do not include every possible technique, they capture the main categories of pharmacological, surgical, mechanical, and non-invasive interventions. If the intervention type were to be the central feature guiding people’s perception of enhancement status, it would indicate that enhancements are synonymous with particular technologies instead of being linked to the goal or riskiness of the procedure.

**Table 1:**
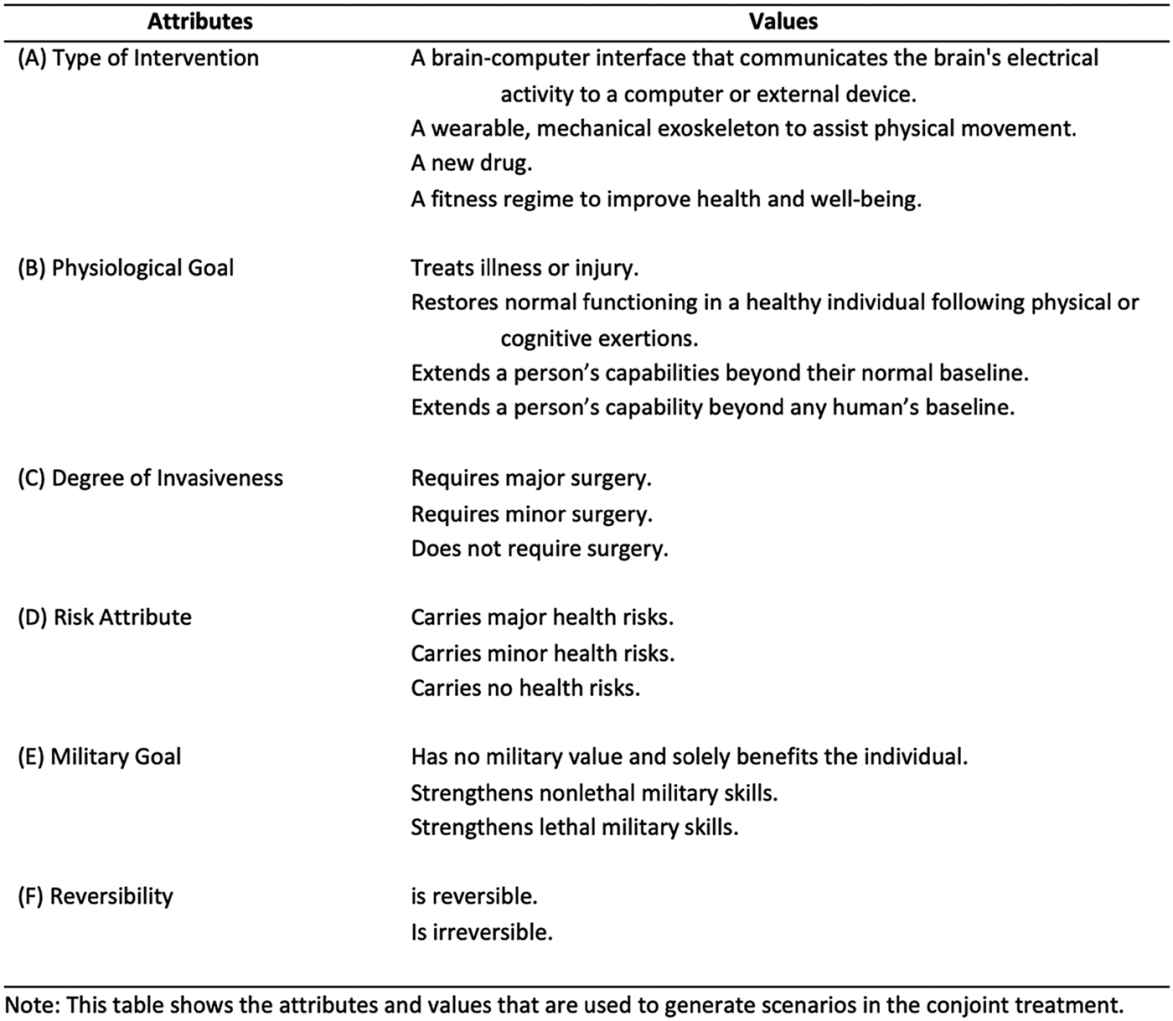
Attributes and values in conjoint treatment

Second, our conjoint scenarios draw from a selection of *physiological goals*. Research has suggested that the motivation of an intervention predicts the way it will be perceived, independent of the technology used (Buchanan, 2011; Menuz et al., 2013; Racine et al., 2021). Therefore, we manipulate whether or not the intervention is designed to treat illness or injury, restore normal functioning in an otherwise healthy person, or extend a patient’s capabilities beyond their or any human’s baseline.

The third attribute relates to the *degree of invasiveness*. We probe the possibility of surgical invasiveness being a key predictor of enhancement perceptions in the expectation that the intrusive properties of the intervention may form a minimal threshold that is required for people to view it as an enhancement (DARPA, 2019).

The fourth feature – *risk attribute* – explores whether the health risks associated with the intervention are predictive of enhancement perceptions. The difference between risk attribute and degree of invasiveness is in the long-term health risks associated with the intervention, compared with the immediate invasiveness.

Fifth, we manipulate the *military goal* of the procedure. For this feature, it is not the methodological features of the intervention that are important, nor the risk for the patient, but the overarching objectives underlying the procedure. This attribute questions whether non-military objectives will still be viewed as ethical enhancement technologies.

Last, we manipulate the *procedure’s reversibility*. An intervention’s reversibility is not necessarily a core feature of enhancements. Think of coffee, which temporarily enhances neurocognitive function before reverting to baseline, or anabolic steroids, which can permanently enhance muscle strength (National Research Council, 2001). Yet separate from the objective facts of enhancement definitions, the *perception* of permanence or reversibility may drive people’s views on whether an intervention constitutes an ethical enhancement technology.

### Sampling Strategy and Survey Design

As one of our aims is to capture the current state of opinion and debate within the epistemic communities where enhancement questions are most salient, we restrict our sampling strategy to professionals who are knowledgeable in this issue-area. Specifically, our inclusion criteria^1^ are limited to those people meeting the following three criteria: (1) academic researchers, scientists, practitioners, government and military officials, or NGO employees who (2) are working on enhancement issues or emerging technologies; and (3) come from fields such as neuroscience, artificial intelligence, bioethics, philosophy, military technology, military science, international law or politics.

In June and July 2024, we received 149 completed responses from within the targeted community. Professionally, 61% of respondents came from academia, 26% from the military, and 13% from government and NGO circles. We obtained a geographically diverse sample, with 49% of respondents residing in North America, 37% from Europe, and 9% from Asia.

A sample size of 149 expert respondents constitutes a meaningful and robust dataset within the context of elite surveys. Surveys targeting high-level experts in ethics, technology, and security often face well-documented challenges: the target population is small, geographically dispersed, disciplinarily varied, and typically difficult to access. For this reason, it is common practice in elite survey research to work with sample sizes in the range of 50 to 200 respondents (López, 2023). We discuss the empirical limitations of this sample in the discussion section. 

After completing a consent form outlining the inclusion criteria, respondents evaluated eight randomly generated scenarios generating 1,192 enhancement assessments. Respondents then completed the enhancement evaluations, and we gathered data relating to psychological predispositions, political attitudes, and demographic details. Information on the scales and measures appears in Online Appendix B.

## Results

**Fig. 2:**
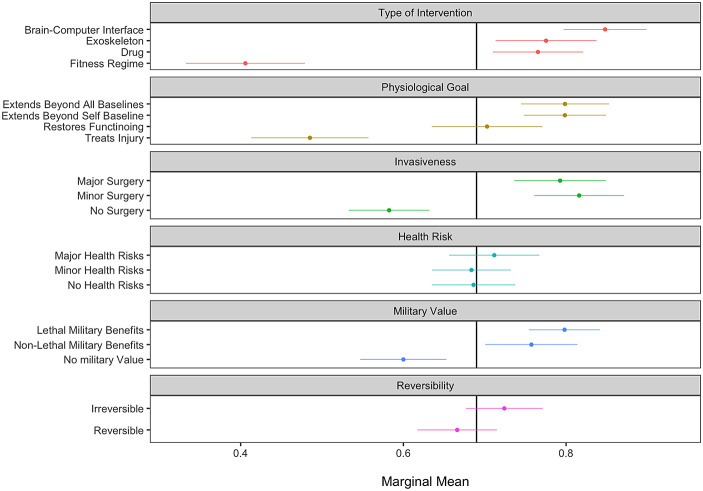
Effects of intervention attributes on its perception as an enhancement technology. Note: This figure displays the marginal mean (MM) estimates for each attribute level, representing the predicted probability that a scenario is classified as an enhancement technology. Estimates are based on the MM model with respondent-clustered standard errors. Bars indicate 95% confidence intervals. The full coefficient table for Fig. 2 appears in Online Appendix C

What features predict whether a respondent classifies a medical intervention as an enhancement technology? Our analytical strategy assesses the relative importance of each attribute by calculating the marginal means (MM) with 95% confidence intervals. MM values describe the proportion of scenarios designated as enhancement technologies when exhibiting a given attribute level (Leeper et al., 2020). In total, respondents designated 69% of scenarios as enhancements. Values that appear to the right of this threshold (marked as the y-axis in Fig. 2) indicate that scenarios featuring the associated attribute have an increased likelihood of being classified as an enhancement. In contrast, values to the left of the y-axis denote the opposite.

Figure 2 displays the MM results showing which attribute values predict enhancement status. Several trends immediately emerge. Noting the effect of the *type of intervention*, we can view a cleavage between pharmacological, surgical, and mechanical interventions compared with a fitness intervention. Nearly 85% of scenarios involving the implantation brain-computer interfaces were viewed as enhancement technologies (SE = 0.02) compared with a mere 41% of interventions involving fitness regimes (SE = 0.04). This attribute has the largest variance of all attributes, which indicates that for many, enhancements are characterized by the nature of the technology and not by the outcomes or risks associated with the interventions. The mere fact that an exoskeleton or BCI appears in a scenario, for example, significantly increases the probability that respondents will classify an intervention as an enhancement, regardless of all other motivational and health implications.

Of the two motive-oriented factors – *physiological goal* and *military value* – we observe small but significant effects. Interventions that extend a person’s baseline capabilities are associated with a larger probability of an intervention being classified as an enhancement However, the magnitude of the extension (beyond self-baseline or beyond all human baselines) does not change this calculus. Likewise, interventions that are designed to create military benefits also extend the likelihood of the procedure being viewed as an enhancement, yet respondents did not distinguish between lethal versus non-lethal benefits. When the outcomes of the interventions had no military benefit and failed to enhance a person’s capabilities beyond a normal baseline, we witnessed a steep reduction in willingness to label the procedure as an enhancement technology. This pattern suggests that military enhancements must be designed to further a military objective, and while this self-referential definition may appear unhelpful, it places a clear emphasis on the objectives of an intervention above the invasiveness of the intervention, to which we now turn.

The last set of predictive factors relates to the riskiness of the procedures—*invasiveness*, *health risks*, and *reversibility*. Respondents placed less emphasis on these factors. Whether procedures were irreversible or involved major health risks did not significantly affect the scenario’s perception as an enhancement. The exception relates to the invasiveness category, where surgical interventions substantially increase the likelihood of a procedure being classified as an enhancement.

**Fig. 3:**
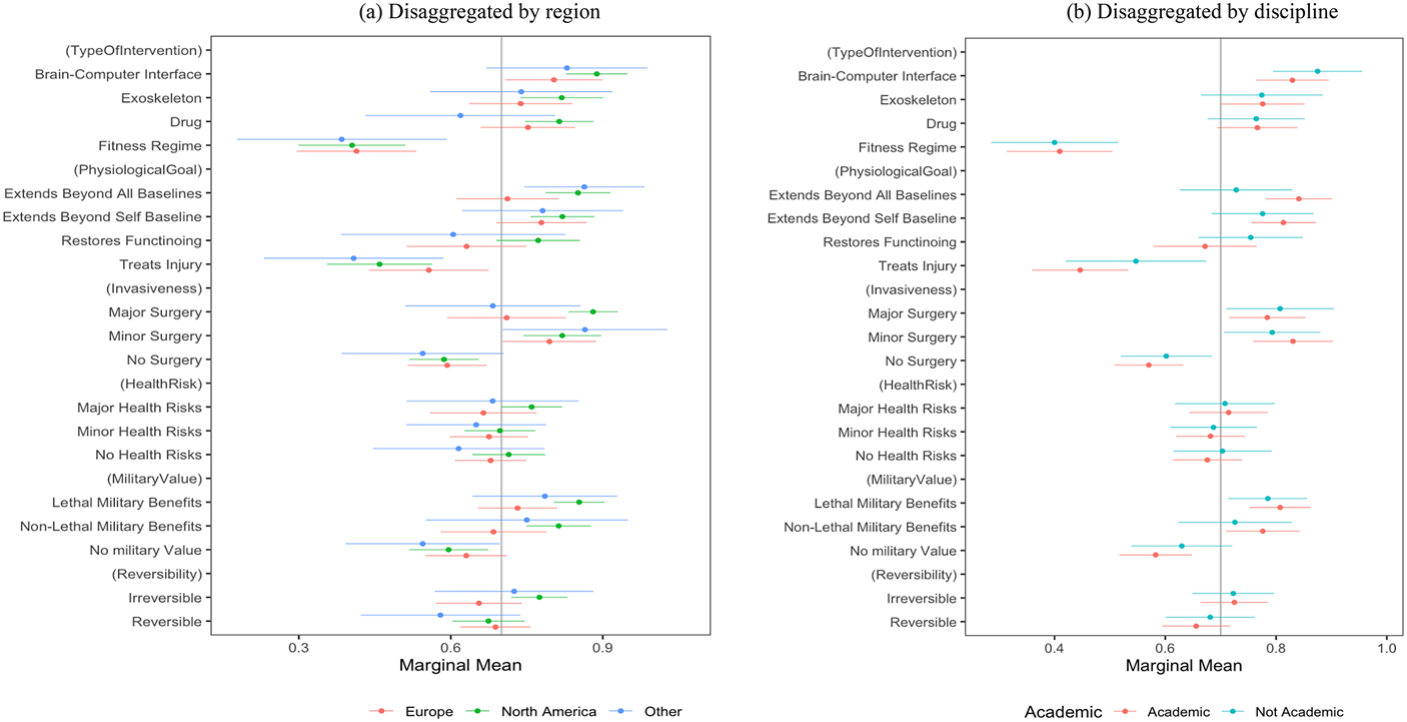
Effects of attributes on perception as enhancement technology, disaggregated by country and discipline. Note: These figures depict estimates of the effects of each randomly assigned attribute value on the probability of a scenario being labeled as an enhancement technology among subgroup populations. Estimates are based on MM models with respondent clustered standard errors. Bars represent 95% CIs

We began by lamenting the lack of consensus about what constitutes enhancement, and it may well be that respondents from different countries reacted differently to our experimental scenarios, or that responses diverged based on various political or psychological factors. As such, we next run a series of interaction analyses to test whether the observed effects are consistent across subgroups of our sample. Figure 3 disaggregates our conjoint results by two variables on which ethical attitudes commonly diverge – region and discipline to see whether disagreements about enhancements are grounded in regional divides, or demonstrate how academics, perched in their ivory towers, depart from the positions of practitioners, and policy and military officials.

Unexpectedly, the results indicate broad agreement about what constitutes enhancement. This is a cross-cutting consensus that spans region, discipline, and, as we show in Online Appendix D, political orientation, gender, age, and military background. To those who proclaim that there are intractable differences that preclude people from coming together to forge a consensus view about how to define enhancement technologies – our findings suggest that a consensus already exists!

**Fig. 4:**
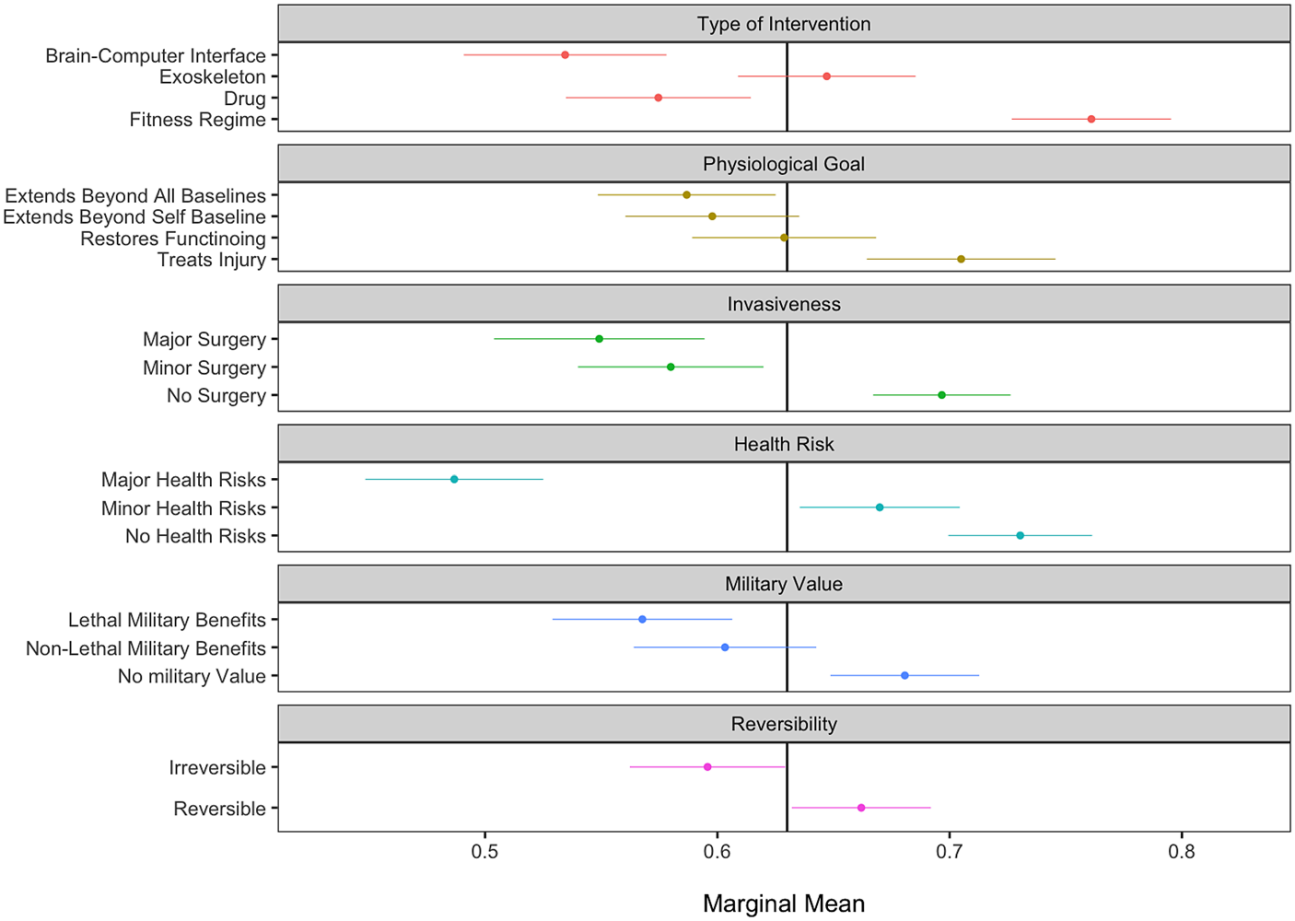
Effects of intervention attributes on its ethical perception. Note: This figure displays the marginal mean (MM) estimates for each attribute level, representing the predicted probability that a scenario is classified as ethical. Estimates are based on the MM model with respondent-clustered standard errors. Bars indicate 95% confidence intervals. Full coefficient table for Fig. 4 appears in Online Appendix E

Turning to the ethics of enhancement, we next asked participants to evaluate the ethical acceptability of each six-attribute based intervention. Figure 4 displays the MM results, showing how each attribute affects an enhancement’s ethical acceptability. Here, we immediately see that the factors predicting that an intervention is ethical are overwhelmingly *the opposite* of those predicting interventions that constitute an enhancement technology. For example, while the involvement of major or even minor surgery heightens the likelihood that an intervention will be viewed as an enhancement, we see that these factors decrease the ethical acceptability of the intervention by 0.58 units on a 0–1 scale (SE = 0.020) and 0.55 units (SE = 0.023) respectively. Brain-computer interfaces and drugs transform interventions into enhancements while dramatically reducing their ethical acceptability, as do interventions that confer lethal military skills, or extend human capabilities beyond all baselines. In contrast, interventions designed to treat injury, decrease their perception as an enhancement technology, while simultaneously increasing their ethical acceptability.

Notably, the *reversibility* attribute had little impact on whether respondents classified an intervention as an “enhancement.” However, it had a substantial effect on ethical evaluations: reversible procedures were significantly more likely to be judged as ethical, while irreversible ones were more often deemed unethical. This divergence suggests that perceptions of enhancement and perceptions of ethical acceptability operate on distinct evaluative axes. Reversibility, in particular, offers a revealing lens into how these dimensions --- often assumed to be diametrically opposed --- are not in fact two sides of the same coin.

## Discussion

Our study demonstrates strong coherence across regions (North America, Europe, and Other) and sectors (academic and non-academic) in what constitutes enhancement. In most cases, demographic background did not substantially influence perceptions of whether an intervention described in a vignette constituted enhancement. This may be because the literature on enhancement has penetrated different regions and sectors in similar ways: while the philosophical literature on enhancement provides divergent definitions, it seems that a norm around this definition does exist.

In some cases, divergence was observed, though not at statistically significant levels for our sample. Whether an intervention was classed as a drug, for example, appeared to generate somewhat divergent perceptions of whether an intervention constituted enhancement between European and non-European respondents; a similar outcome was observed when interventions involved major surgery. This may be explainable through the priming cases that contemporary work on military enhancement has used across the Atlantic. Europeans, for example, have focused more on exoskeletons and other forms of exogenous augmentation as enhancements than North Americans, where the latter tend to focus extensively on pharmacological interventions (Julienne, 2022; Brunyé et al., 2020; National Research Council, 2009).

An important consequence of this study is that future policy may be able to accommodate a definition of enhancement that is widely shared among members of the international community. A recent strategy released by NATO, for example, defines “Human Enhancement Technologies [as] biotechnological and non- biotechnological interventions that enable individuals to operate beyond normal human limits or abilities,” and this definition, without much additional effort, may be acceptable at an international level to define the scope of future military enhancement policy and norms (NATO, 2024).

### Is Enhancement Ethical?

Our results highlight an inverse relationship between a technology’s classification as an enhancement and its degree of ethical acceptability in the eyes of experts. The more that respondents agree that a technology qualifies as an enhancement, the less they think that the same technology is ethically acceptable. The most robust examples of enhancement are pharmaceutical (drugs), mechanical (exoskeleton), and neural (BCI) military interventions that push individuals beyond normal baselines. These might include Modafinil to counter fatigue and increase endurance and stamina, full-body or partial-body exoskeletons, wearable or surgically implanted prosthetics to increase strength dramatically, and wearable or implanted brain-computer interfaces linking warfighters with weapons to improve targeting accuracy and response rates (Van Puyvelde et al., 2022; Walker & Sparrow, 2024; Billing et al., 2021). In contrast, the least robust enhancements are technologies designed for therapeutic purposes that improve or restore health following illness and injury. Medical interventions, such as prosthetic rehabilitation following limb amputation, are among the most ethically acceptable but do not fully qualify as enhancements.

While therapeutic medical interventions often require major surgery and incur risk, these risks are morally acceptable when offset by improved health benefits for the individual who receives medical treatment. However, the purported advantages of pharmaceutical and neural military enhancements do not similarly offset these costs because they primarily benefit military capabilities, not the enhanced individual. The enhanced warfighter incurs personal risks to gain physical strength or cognitive acumen of great use to the military but of little, if any, personal benefit. For these reasons, our data underscore reservations about surgically invasive and medically risky military enhancement. However, the data push further, highlighting the potential pitfalls of any technologically sophisticated human-extending *military* enhancement regardless of surgical invasiveness and medical risk.

As we evaluate the ethical acceptability of enhancement, it is crucial to emphasize that our data do not equate diminished ethical acceptability with an unacceptable or impermissible enhancement technology. Returning to Fig. 4, a score of 0.5 is the exact midpoint between ethically acceptable and unacceptable, so a score of 0.4 is somewhat unacceptable, and 0.6 is somewhat acceptable. This places such enhancements as drugs, implanted exoskeletons, and BCIs that push warfighters beyond normal baselines for military purposes close to or just beyond the midpoint, an area we describe as morally precarious rather than firmly unacceptable. Caution is the watchword.

Pharmaceutical and neural military enhancements are precarious because they tread on two morally contentious grounds: experimental medical research and war. Except for several unique precautions surrounding the enlistment of military personnel for medical experimentation (e.g., a designated ombudsman), the ethical principles governing volunteer medical research as subjects are largely the same in civilian and military contexts (Gross, 2021). In both cases, informed consent, patient safety, and the expectation of medical benefits guide medical research. Fulfilling these conditions, enhancement research earmarked for therapeutic purposes is ethically robust.

These conditions are necessary but insufficient when enhancement is designed for military purposes. Further conditions draw attention to their military use and purpose. Concerns arise about soldiers’ consent to participate in research or use enhancements, the disparate capabilities among the ranks as the result of enhancements, unfair allocations of new technologies, and discharged soldiers’ reintegration into civil society. Failure to address these concerns raises moral hazards that call for oversight, supervision, and recourse for soldiers who want to opt-out or encounter problems with their enhancement (Parasidis, 2016; European Parliament, 2014; McManus et al., 2007).

The second military challenge concerns the ethics of war. When an enhancement has therapeutic value, ethical issues will rarely arise unless the technology is used for unlawful purposes or distributed egregiously and unjustly. Therapeutic enhancements are *ipso facto* ethical because they improve human health. In contrast, improving warfighting capabilities is not inherently ethical unless accompanied by a just war and just warfighting. Just war includes wars of national self-defense to stave off aggression and humanitarian intervention to combat grave human rights abuses. Just war fighting entails protections for soldiers and civilians. Soldiers are protected from torture and inhumane harm, while civilians are protected from unnecessary and disproportionate death and injury. Many observers raise concerns that enhanced warfighters may disregard these protections as they single-mindedly pursue their military mission (Liivoja, 2022; Dinniss & Kleffner, 2018). Pinpointing the injustices of war is always vexing and requires mechanisms for incorporating legal guidelines into military operations, judicial oversight, and enforcement at the national and international levels.

Acknowledging that enhancement research, development, and deployment must fulfill these many conditions addresses the ethical perils of pharmaceutical and neural military enhancements. Still, their road to ethical acceptance is far more arduous than therapeutic technologies must travel. Our data, therefore, offer a wake-up call to exercise extreme caution for all kinds of sophisticated military enhancements. While some nations, such as France, are prepared to severely restrict pharmaceutical neural military research, many other national authorities push for strict oversight (Girling et al., 2017; de Boisboissel & Revue, 2019; UK Ministry of Defense, 2021; UK Department of Defense, 2020). The current literature, too, suggests that many nations are angling for prudence, greater oversight, independent review, and transparent discourse.

### Enhancement and the Adverse Relationship Between Medicine and War

This study adds another page to the historically adverse and tenuous relationship between medical technology and war. Following WWII, the British research laboratory at Porton Down segregated its scientists developing chemical and biological weapons (CBW) by assigning physicians (MDs) to defensive projects and the remaining specialists (PhDs) to its offensive projects. The reasoning was simple: medicine is about saving lives; war is about taking them. Tasking physicians to develop bombs put many ill at ease. Addressing emerging weapons technology in 1997, the International Committee of the Red Cross (ICRC) hoped to limit weapons that cause a “specific disease, a specific abnormal physiological state, specific and permanent disability or specific disfigurement” (Coupland, 1996, 1999). All four outcomes suggest medical malfeasance, potential violations of humanitarian law, and cause for extreme caution when weaponizing medicine.

Our data suggests that enhancement technology presents similar discomfort. Military enhancement, the gold standard of human capacity-extending enhancement, is paradigmatic. Among the attributes surveyed, a surgically implanted medical technology that restores or extends human functioning for military purposes is archetypical and the most morally contentious. Here, too, medicine and war collide. Enhancement exploits medical technology to weaponize warfighters through abnormal physiological states that so alarmed the ICRC. How, then, should performance enhancement technologies move forward?

Maintaining a bright line between therapeutic and non-therapeutic enhancements is one answer. North of the line, it’s easygoing; south of the line, it is grueling. But the line is not neat, particularly when factoring in medicine’s contribution to war. Medicine may contribute to war by caring for injured soldiers or boosting military capabilities. Caring for the sick and injured signals therapeutic medicine whether soldiers return to military duty or civilian life. In each case, medicine does its best to restore some semblance of normal functioning. Although medicine serves war by maintaining combat readiness and returning some soldiers to combat, these interventions are not ethically contentious unless nations wage war unlawfully.

In contrast, technologies that extend human capacities well beyond human limits are the most contentious, particularly when used to enhance warfighting skills. These technologies place medicine in the service of war directly. Invasive technologies such as BCIs, bionic prosthetics (i.e., surgically implanted exoskeletons), and pharmaceuticals create abnormal physiological states and are, according to our data, the most morally precarious. Yet, enhancement technologies may indirectly serve war by improving education and training for war-supporting services (e.g., administration, logistics, and supply) rather than warfighting capabilities.

Where the medical community fits in is still unresolved. It is tempting to confine medicine to human health and avoid contributions to warfighting unrelated to health. But this is not always practical. While it was tempting to segregate scientists by professional affiliation (MD or PhD), the line between defensive and offensive research broke down early. Medical scientists might develop vaccines for biowarfare, but it was a short step to genetically modify their experimental viruses for offensive purposes. Similarly, medical enhancement may try to stop short of transhumanism and refuse to develop technologies that bolster war-fighting performance. But faced with the exigencies of war and the demands of military necessity, researchers may easily push interventions past the “normal baseline” by altering doses or electro-mechanical configurations, for example. Used as intended or not, medical technologies recruited for war pose constant challenges. The first, highlighted in this study, bids practitioners to call out ethical precariousness. With that, comes the challenge to exercise caution and sustain dialogue, transparency, and oversight in during peace and war.

While we have established convergence among our sample, our results do not establish the granular reasons behind these judgements. Convergence of this kind, at a policy level, is often underscored by divergent substantive reasons between parties. This has been a frequent concern, for example, in human subjects research, where different conceptions of the appropriate subject of research, balance of risk and benefits, and basis of informed consent give way to common sets of rules (in the US, *the* “Common Rule”) for undertaking human research—rules that have recently been suggested are capable of accommodating research into military enhancement technologies (Evans et al.,^ 2024^).

Future empirical work will be required to understand how, and when, individuals come to common conclusions about the ethics of military enhancement in practice. We do not claim to have uncovered a universal consensus, but rather to have revealed a stronger-than-expected degree of convergence across a diverse, interdisciplinary sample of experts. We view this as a useful empirical contribution—an initial step toward clarifying conceptual boundaries and fostering deeper dialogue.

Going forward, qualitative measures might be employed among expert samples such as ours to understand, given future military decision-making around enhancement, what kinds of values translate to convergence of the kind we describe here. Future work should also periodically re-sample the expert community to examine whether attitudes shift in response to new technological developments or evolving normative frameworks; we are committed to this latter kind of endeavor. These future surveys will also address current gaps by engaging underrepresented and missing communities in the current sample. We welcome others to join us in advancing this conversation.

## Supplementary Information

Below is the link to the electronic supplementary material.

Supplementary Material 1

## Footnotes

1 See Online Appendix A for a detailed review of our recruiting strategy and sample statistics.
