# Supplementary material for: Enhancing Soldiers for Future Warfare: Good Science; Bad Ethics?
Source: Sci Eng Ethics. 2025 Dec 15;32(1):5. doi: 10.1007/s11948-025-00573-w (PMC12799693; doi:10.1007/s11948-025-00573-w)
Supplement: Supplementary file 1 — Supplementary Material 1 [file 11948_2025_573_MOESM1_ESM.docx]

Online Appendix

**Enhancing Soldiers for Future Warfare: Good Science; Bad Ethics?**

[Appendix A: Recruiting Strategy and Sample Statistics A1](#_Toc183947204)

[Appendix B: Codebook of Measures Used in Questionnaire A2](#_Toc183947205)

[Appendix C: Results for Enhancement Definition Analyses A3](#_Toc183947206)

[Appendix D: Results for Enhancement Interaction Analyses A4](#_Toc183947207)

[Appendix E: Results for Enhancement Ethics Analyses A7](#_Toc183947208)

# **Appendix A: Recruiting Strategy and Sample Statistics**

A distinguishing feature of our study is the decision to sample elite respondents. There exists considerable diversity of perspective about what constitutes military enhancement among scientists, ethicists and military personnel. Seeing as one of our aims is to capture the current state of opinion and debate within the epistemic communities where enhancement questions are most salient, we restrict our sampling strategy to those members of the community with recognized knowledge in this issue-area. It is from this community that a position on enhancement must emerge. Specifically, our inclusion criteria are limited to people who meet the following three criteria:

1. Academic researchers, scientists, practitioners, government and military officials, or NGO employees; and
2. Who are working on enhancement issues or emerging technologies; and
3. Come from fields such as neuroscience, artificial intelligence, bioethics, philosophy, military technology, military science, international law or politics.

We employed a snowball sampling method to attain qualified respondents. We began by assembling an initial list of scientists and government / military officials who had participated in a seminal 2023 workshop dedicated to the ethics of enhancements. To supplement this list and add geographical and disciplinary diversity to our sample, we collated a list of 723 published academic articles on the topic of bioethics and enhancement technologies. Finally, we reached out to the membership of scientific communities, government organizations, and NGOs related to ethics, enhancement, and emerging technologies.^[[1]](#footnote-1)^

In total, we received 149 completed responses from within the targeted community. Professionally, 61% of respondents came from academia, 26% were from the military, and 13% came from government and NGO circles. We obtained a geographically diverse sample, with 49% of respondents residing in North America, 37% from Europe, and 9% from Asia. The “price” that we pay for surveying an elite epistemic community is that the demographics of our sample are not representative of the broader population. Instead, they are representative of the standard profile of members of the community. Accordingly, 92% of our sample possess a graduate degree, a fact that reflects the educational norms of membership in this community. 78% of our sample were male, and the median age was 51. Politically, 71% of survey respondents self-identified as left-wing, reflecting the ideological homogeneity within this community.

# **Appendix B: Codebook of Measures Used in Questionnaire**

| **Variable Name** | **Variable Construction** |
| --- | --- |
| Risk Aversion | Six-item measure asking participants about their level of agreement with statements that capture risk aversion. Items include: “I do not feel comfortable about taking chances” and “Before I make a decision, I like to be absolutely sure how things will turn out.” Items were answered on a scale of 1 (strongly disagree) to 6 (strongly agree). Final value is a mean of the six items. Scale sourced from Mandrik & Bao (2005). |
| Baseline Enhancement Views | Two-item measure asking whether participants believe that the following examples should be considered a form of enhancement: “Lifestyle interventions such as better exercise, sleep, and mindfulness” and “Attaining an ivy league education”. Items were answered on a scale of 1 (not at all) to 5 (absolutely). |
| Enhancement Status | Our first dependent variable. Participants registered their belief about whether the randomly generated conjoint scenario described an enhancement technology. Scenarios were assessed as a binary outcome indicating that it is or is not an enhancement technology. |
| Ethical Status | Our second dependent variable. Participants registered their belief about whether the randomly generated conjoint scenario is ethically acceptable. Scenarios were assessed on a scale of 1 (absolutely unacceptable) to 6 (absolutely acceptable). |
| Age | Participants recorded their year of birth. |
| Gender | Offered a selection of male, female, non-binary, or prefer not to say. |
| Education | Participants were asked what is the highest level of education they had completed? Nine options were provided ranging from less than high school, up to advanced graduate degree. The final product was transformed into a four-point scale. |
| Employment | Participants were asked about their field of work. Six options were provided (along with an other option), which reflected the fields targeted as part of our recruitment strategy (academia, military, government, NGOs, etc.) |
| Political Orientation | Single-item measure asking respondents: “How would you describe your political position on a scale from extremely liberal (1) to extremely conservative (6)?” |
| Region | Participants were asked to record their region of residence. |
| Military service | Participants were asked whether they had ever served in the armed forces, and if so, whether they had served in a combat role. |
| Pop Culture | Participants were asked on a four-point scale whether now, or when they were growing up, they consumed science fiction popular media. As a follow-up measure, participants marked their familiarity with 16 different science-fiction titles (i.e., Captain America, Halo, Battlefield Galactica). |
| Medical History | Without asking for medical details, participants recorded whether or not they have chronic medical conditions, whether they’ve had to undergo surgery, and whether have suffered a medical injury that required physical rehabilitation |

# **Appendix C: Results for Enhancement Definition Analyses**

In Figure 2, in the main manuscript, we display the marginal means results that reveal which attribute values predict whether respondents view a scenario as an enhancement technology. Table C1 lists the fully specified conjoint table with the precise coefficient estimates and standard errors for each item.

**Table C1: Marginal Mean Conjoint Estimates**

| **Attribute** | **Level** | **Estimate** | **Std. Err** |
| --- | --- | --- | --- |
| Type Of Intervention | Fitness Regime | 0.4058 | 0.0373 |
| Type Of Intervention | Drug | 0.7655 | 0.0283 |
| Type Of Intervention | Exoskeleton | 0.7754 | 0.0317 |
| Type Of Intervention | Brain-Computer Interface | 0.8480 | 0.0260 |
| Physiological Goal | Treats Injury | 0.4851 | 0.0367 |
| Physiological Goal | Restores Functioning | 0.7029 | 0.0347 |
| Physiological Goal | Extends Beyond Self baseline | 0.7985 | 0.0258 |
| Physiological Goal | Extends Beyond All Baselines | 0.7987 | 0.0276 |
| Invasiveness | No Surgery | 0.5825 | 0.0253 |
| Invasiveness | Minor Surgery | 0.8161 | 0.0282 |
| Invasiveness | Major Surgery | 0.7926 | 0.0288 |
| Health Risk | No Health Risks | 0.6863 | 0.0261 |
| Health Risk | Minor Health Risks | 0.6837 | 0.0248 |
| Health Risk | Major Health Risks | 0.7117 | 0.0283 |
| Military Value | No Military Value | 0.6000 | 0.0271 |
| Military Value | Non-Lethal Military Benefits | 0.7574 | 0.0289 |
| Military Value | Lethal Military Benefits | 0.7981 | 0.0224 |
| Reversibility | Reversible | 0.6661 | 0.0249 |
| Reversibility | Irreversible | 0.7241 | 0.0241 |

*Note: All tests are two-tailed, all standard errors are clustered at the respondent level.
Signif. Codes: *: 0.05, **: 0.01, ***: 0.001*

# **Appendix D: Results for Enhancement Interaction Analyses**

In Figure 3, we run an interaction analysis revealing that there are only minor differences in the way that our conjoint items predict enhancement status when comparing between participants in different regions (North America, Europe, other) and by discipline (academic, non-academic). In the following tables, we demonstrate that the same cross-cutting agreement persists when we subset the results by other covariates such as political orientation, gender, age, and military background.

These results reinforce the notion that there exists a clear consensus as to which forms of medical interventions constitute an enhancement technology. Despite this consensual pattern, we point to the following areas where subgroup differences exist. First, we witness a clear age divide when it comes to the effect of major surgery. Older survey respondents are more likely to view scenarios involving major surgery as constituting an enhancement technology than younger participants. We expect that this is due to the fact that older people have undergone major surgery more often than younger people, hinting that people look to their own medical experiences in deciding what is an enhancement. Second, we witness a small gender divide in relation to one attiribute, where women are more certain that brain-computer interfaces constitute an enhancement technology than men holding all other variables constant.

**Figure D1: Effects of intervention attributes on perceptions of enhancement technology, disaggregated by partisanship and gender**

(a) Disaggregated by political orientation (b) Disaggregated by gender


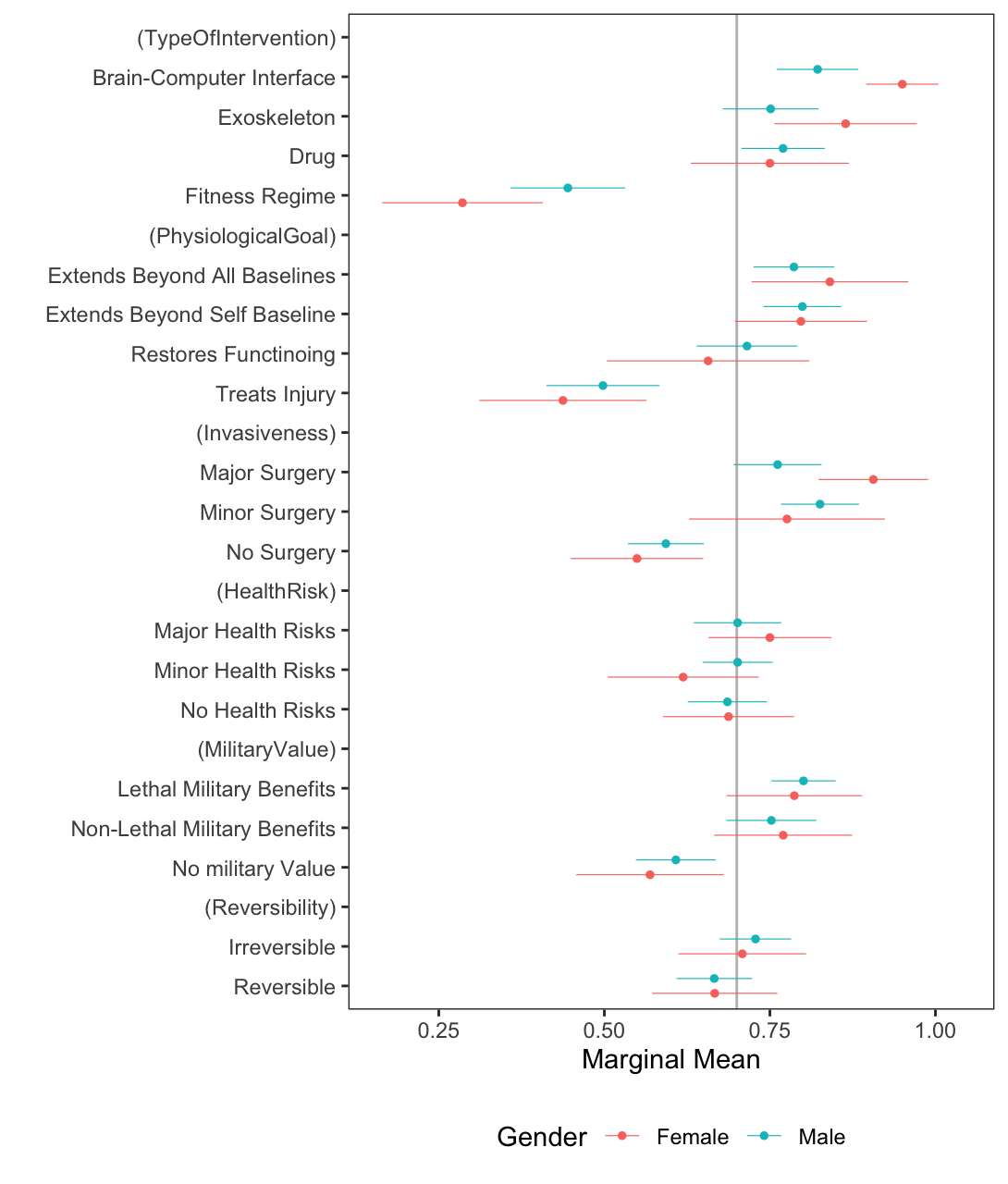

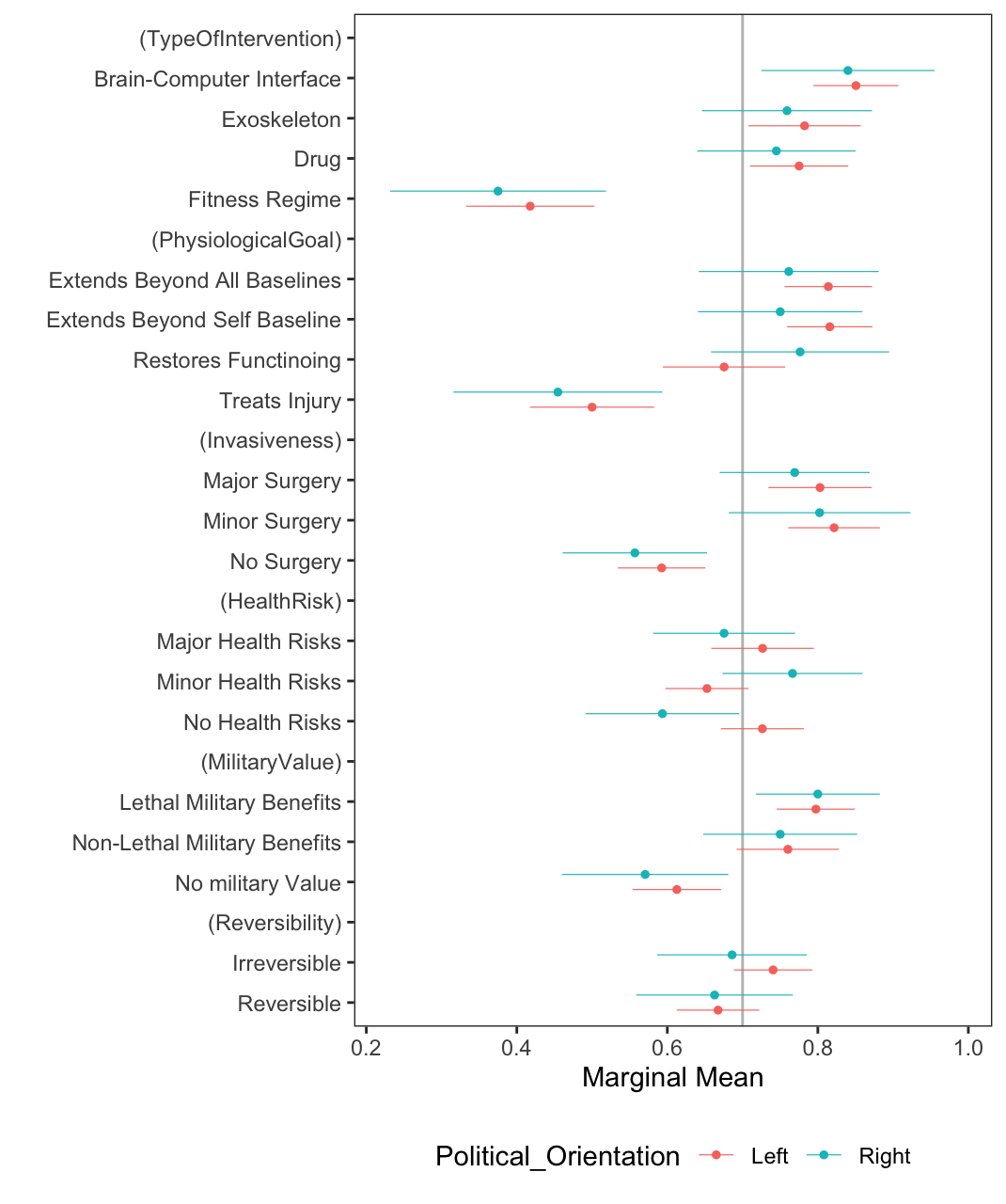


**Figure D2: Effects of intervention attributes on perceptions of enhancement technology, disaggregated by military background and age**


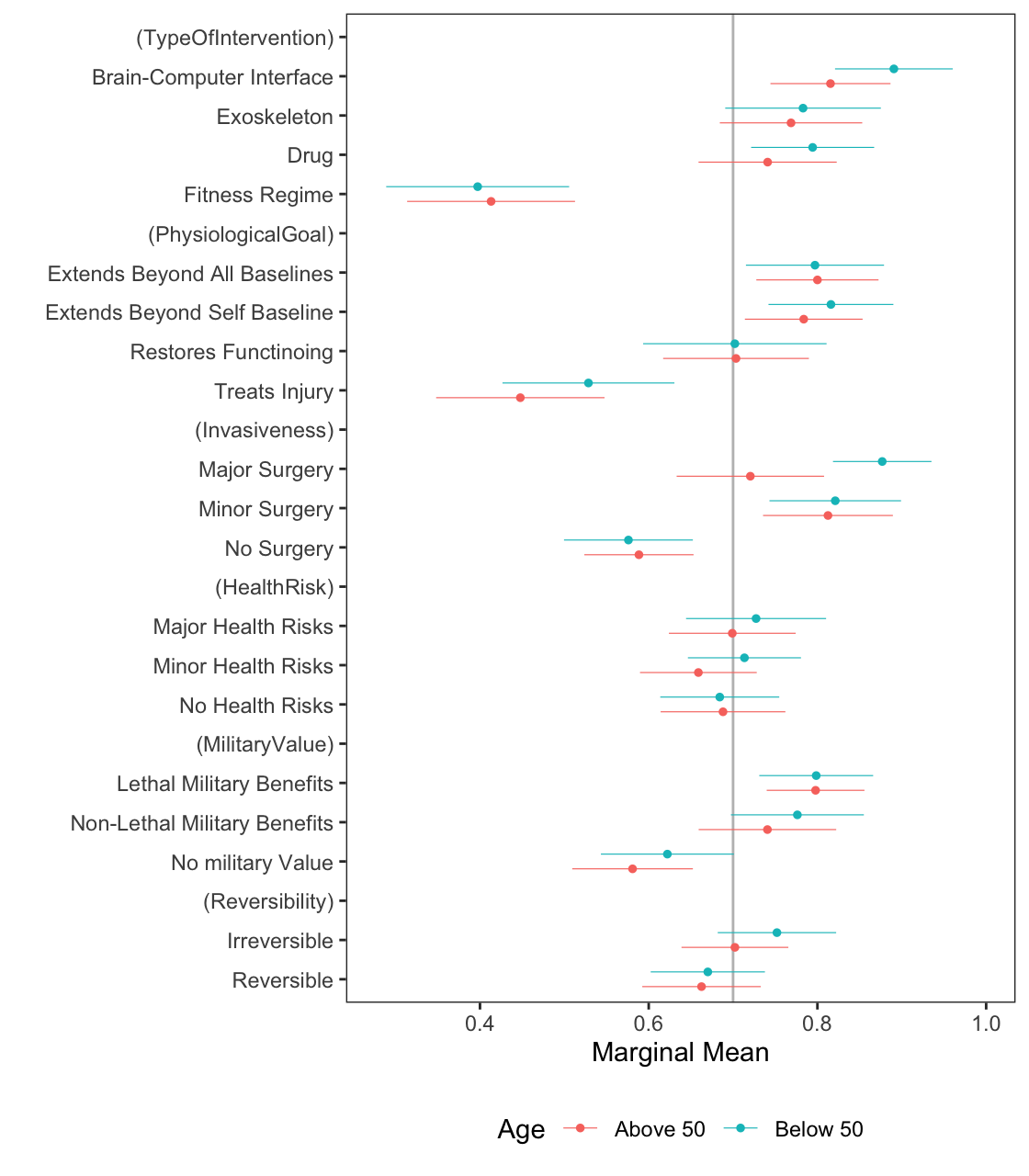

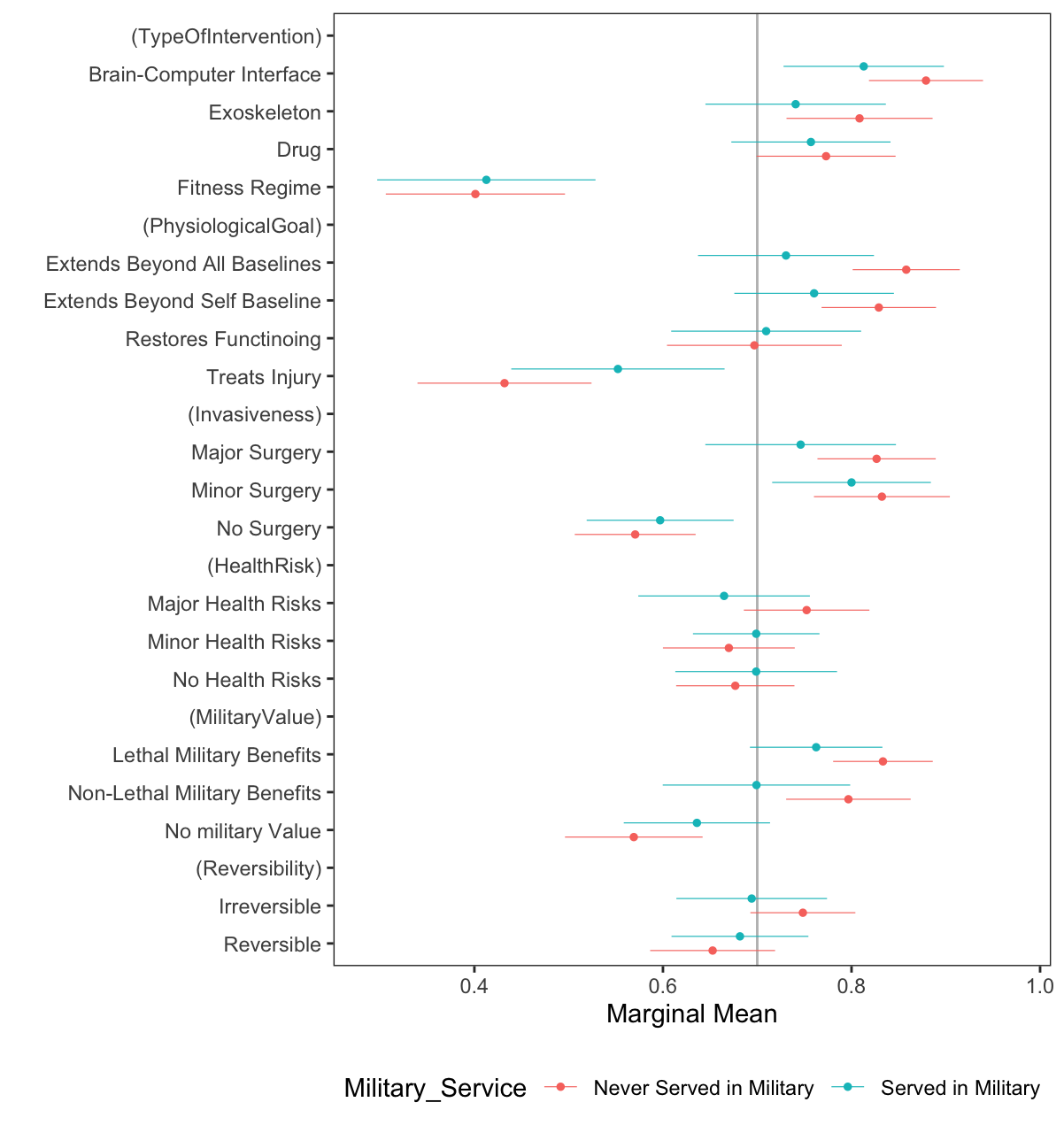
 (a) Disaggregated by military background (b) Disaggregated by age

# **Appendix E: Results for Enhancement Ethics Analyses**

In Figure 4, in the main manuscript, we display the marginal means results that reveal which attribute values predict whether respondents view a scenario as an ethically acceptable. Table E1 lists the fully specified conjoint table with the precise coefficient estimates and standard errors for each item.

**Table E1: Marginal Mean Conjoint Estimates**

| **Attribute** | **Level** | **Estimate** | **Std. Err** |
| --- | --- | --- | --- |
| Type Of Intervention | Fitness Regime | 0.7610 | 0.0175 |
| Type Of Intervention | Drug | 0.5746 | 0.0203 |
| Type Of Intervention | Exoskeleton | 0.6471 | 0.0195 |
| Type Of Intervention | Brain-Computer Interface | 0.5345 | 0.0223 |
| Physiological Goal | Treats Injury | 0.7050 | 0.0207 |
| Physiological Goal | Restores Functioning | 0.6288 | 0.0202 |
| Physiological Goal | Extends Beyond Self baseline | 0.5978 | 0.0191 |
| Physiological Goal | Extends Beyond All Baselines | 0.5868 | 0.0195 |
| Invasiveness | No Surgery | 0.6966 | 0.0151 |
| Invasiveness | Minor Surgery | 0.5799 | 0.0204 |
| Invasiveness | Major Surgery | 0.5492 | 0.0231 |
| Health Risk | No Health Risks | 0.7304 | 0.0158 |
| Health Risk | Minor Health Risks | 0.6699 | 0.0176 |
| Health Risk | Major Health Risks | 0.4867 | 0.0195 |
| Military Value | No Military Value | 0.6807 | 0.0163 |
| Military Value | Non-Lethal Military Benefits | 0.6033 | 0.0200 |
| Military Value | Lethal Military Benefits | 0.5677 | 0.0197 |
| Reversibility | Reversible | 0.6620 | 0.0153 |
| Reversibility | Irreversible | 0.5958 | 0.0171 |

*Note: All tests are two-tailed, all standard errors are clustered at the respondent level.
Signif. Codes: *: 0.05, **: 0.01, ***: 0.001*

1. For example, we reached out to members of ISME, the International Society of Military Ethics. [↑](#footnote-ref-1)
